# Pharmacogenetics of Statin-Induced Myotoxicity

**DOI:** 10.3389/fgene.2020.575678

**Published:** 2020-10-16

**Authors:** Ping Siu Kee, Paul Ken Leong Chin, Martin A. Kennedy, Simran D. S. Maggo

**Affiliations:** ^1^Gene Structure and Function Laboratory, Carney Centre for Pharmacogenomics, Department of Pathology and Biomedical Science, University of Otago, Christchurch, New Zealand; ^2^Department of Medicine, University of Otago, Christchurch, New Zealand

**Keywords:** statins, pharmacogenetics, pharmacogenomics, muscle toxicity, myotoxicity, adverse effects, SLCO1B1

## Abstract

Statins, a class of lipid-lowering medications, have been a keystone treatment in cardiovascular health. However, adverse effects associated with statin use impact patient adherence, leading to statin discontinuation. Statin-induced myotoxicity (SIM) is one of the most common adverse effects, prevalent across all ages, genders, and ethnicities. Although certain demographic cohorts carry a higher risk, the impaired quality of life attributed to SIM is significant. The pathogenesis of SIM remains to be fully elucidated, but it is clear that SIM is multifactorial. These factors include drug–drug interactions, renal or liver dysfunction, and genetics. Genetic-inferred risk for SIM was first reported by a landmark genome-wide association study, which reported a higher risk of SIM with a polymorphism in the *SLCO1B1* gene. Since then, research associating genetic factors with SIM has expanded widely and has become one of the foci in the field of pharmacogenomics. This review provides an update on the genetic risk factors associated with SIM.

## Introduction

**FIGURE 1:**
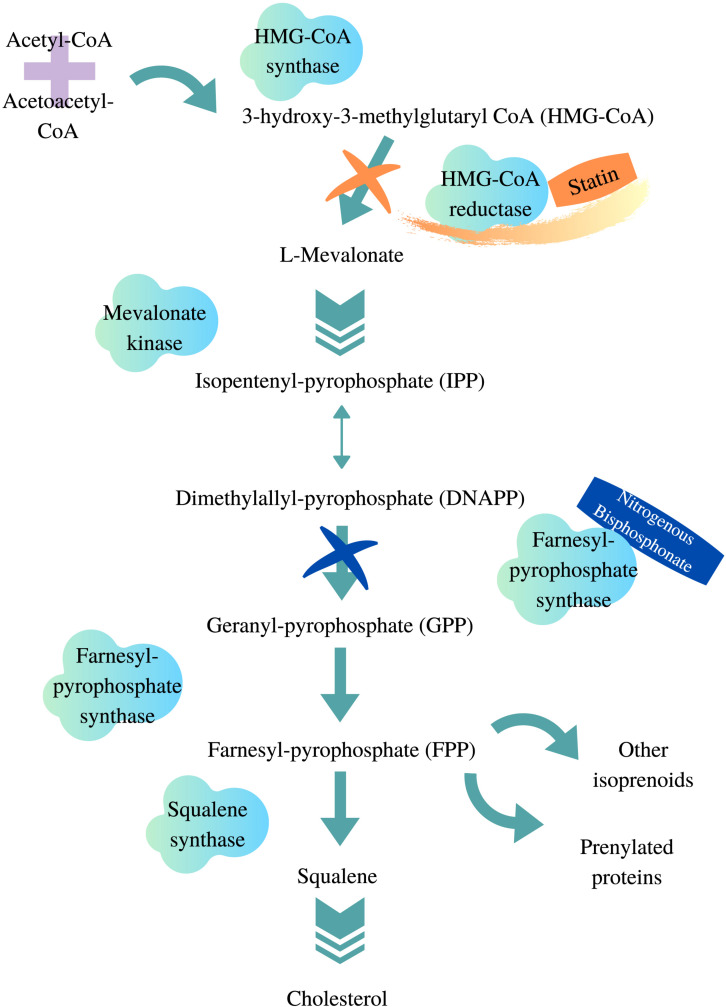
HMG-CoA reductase pathway. Adapted from van Beek et al. (2003), Gazzerro et al. (2012), designed with Canva.com.

Hydroxymethylglutaryl-coenzyme A reductase (HMG-CR) inhibitors, commonly known as statins, are indicated for hypercholesterolemia and atherosclerotic cardiovascular diseases (Grundy et al., 2019; Bartlomiejczyk et al., 2019). The pharmacological effects of statins include reducing blood plasma concentrations of total cholesterol, triglycerides, low-density lipoprotein (LDL) cholesterol, as well as increasing the level of high-density lipoprotein cholesterol (Sizar et al., 2019). Statins bind competitively to the HMG-CoA reductase enzyme and inhibit the endogenous production of cholesterol within hepatocytes (Figure 1). This reduces endogenous cholesterol levels, which in turn upregulates LDL receptors on the cell surface of hepatocytes, resulting in the removal of circulating cholesterol in the blood (Brown and Goldstein, 1981; Young and Fong, 2012). The first statin discovered was mevastatin, which was isolated from a fungus by Akira Endo in the 1970s (Endo, 2010). Since then, statins have been of interest to both academic and pharmaceutical fields, which has led to the discovery and synthesis of various statins available clinically today. While all statins share the same mechanism of action, they exhibit very different pharmacokinetic (PK) properties.

To date, the only Food and Drug Administration (FDA)–approved route of administration for statins is oral (Korani et al., 2019). Generally, statins are rapidly absorbed following administration, reaching peak plasma concentration within 4 h in immediate-release formulations (Wiggins et al., 2016). All statins are administered in active hydroxy acid forms, except simvastatin and lovastatin, which require *in vivo* biotransformation from their lactone prodrug forms to exert pharmacological effects (Vickers et al., 1990; Vyas et al., 1990a; Taha et al., 2016). Based on their solubility, statins are transported systemically either through passive diffusion or actively assisted by endogenous transporters such as the adenosine triphosphate (ATP)–binding cassette (ABC) and solute carrier (SLC) transporters (Whirl-Carrillo et al., 2012). The liver is the site of action for statins, as well as for their metabolism. Some statins such as simvastatin (Kyrklund et al., 2000) and lovastatin (Zhu et al., 2011) also undergo intestinal degradation. Generally, statins are metabolized in hepatocytes prior to elimination via bile (Whirl-Carrillo et al., 2012). Cytochrome P450 (CYP450) enzymes are mainly responsible for oxidative biotransformation of statins (Brown et al., 2008), whereas conjugation through glucuronidation is commonly facilitated by the uridine 5′-diphospho-glucuronosyltransferase (UGT) family of enzymes (Schirris et al., 2015; Sanchez-Dominguez et al., 2018).

**FIGURE 2:**
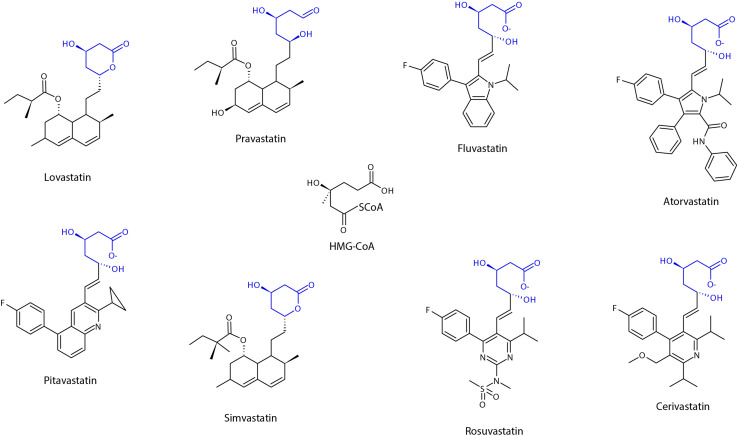
Chemical structure of statins, showing the shared pharmacophore of HMG-CoA. Adapted from Schachter (2005), designed with Chemsketch Version 2019.2.2 (C05E41) (ACD/Labs), Toronto, Ontario Canada.

Structurally, all statins share a similar pharmacophore with the HMG-CR moiety as shown in Figure 2 (Arnaboldi and Corsini, 2010). Nonetheless, their interactions with the pocket binding site of HMG-CR enzyme vary, which contributes to their different potencies as HMG-CR inhibitors (Arnaboldi and Corsini, 2010). Among the statins available for prescription, pitavastatin is the most potent. However, rosuvastatin and atorvastatin achieved the greatest effect in lowering LDL cholesterol for the recommended dose range (Adams et al., 2020). In addition, the PK properties of statins also vary (Schachter, 2005). The different PK properties and transporter substrate specificity of statins are summarized in Table 1.

**TABLE 1:** Pharmacokinetic profile of statins [adapted and compiled from Bischoff et al. (1997), Hu et al. (2009), Sirtori et al. (2012), Maji et al. (2013)].

Parameter	Lovastatin	Simvastatin	Pravastatin	Fluvastatin	Atorvastatin	Cerivastatin	Rosuvastatin	Pitavastatin
Generation	First	Second	First	First	Second	Withdrawn	Third	Third
Potency (nM)*	2–4	1–2	4	3–10	1.16	1	0.16	0.1
Solubility	Lipophilic	Lipophilic	Hydrophilic	Lipophilic	Lipophilic	Lipophilic	Hydrophilic	Lipophilic
Oral absorption (%)	30	60–85	35	98	30	> 98	50	80
Protein binding (%)	> 98	>95	50	> 98	>98	> 99	90	96
Bioavailability (%)	5	< 5	18	30	12	60	20	60
Hepatic extraction (%)	≥ 70	≥80	45	≥ 70	70	50–60	63	N/A
Half-life (h)	2–5	2–5	1–3	1–3	7–20	1–3	20	10–13
Renal excretion (%)	10	13	20	6	< 5	30	10	N/A
CYP450 metabolism	3A4/3A5 (2C8?)	3A4/3A5 (2C8, 2D6)	(3A4)	2C9	3A4 (2C8)	3A4, 2C8	2C9 (2C19)	(2C9)
SLC transporter substrate	SLC01B1	SLCO1B1	SLC01B1/2B1	SLCO1B1	SLCO1B1	SLCO1B1	SLCO1B1/1B3/ 2B1/1A2,SLC10A1	SLCO1B1/ 1B3
ABC transporter substrate	ABCB1	ABCB1/C2	ABCB1/B11/ C2/G2	ABCG2	ABCB1/G2	ABCB1/C2/G2	ABCB1/C2/G2	ABCB1/C2/ G2

*Measured as IC_50_ (concentration of inhibitor to produce 50% of inhibition). Content in parentheses indicates a minor metabolic pathway.

Statins, while generally considered to be a well-tolerated class of drugs, are associated with adverse effects. A systematic review and meta-analysis of randomized trials with more than 90,000 participants showed that statins significantly increased the relative and absolute risk of myopathy, renal dysfunction, and hepatic dysfunction (Yebyo et al., 2019). Statin-induced myotoxicity (SIM) and hepatotoxicity are common adverse effects with prevalence rates ranging from 7 to 30% and 2 to 5%, respectively (Bjornsson, 2017; du Souich et al., 2017). They were shown to be dose-dependent (McClure et al., 2007; Raju et al., 2013; Bjornsson, 2017). Unlike myotoxicity and hepatotoxicity, renal dysfunction associated with statin use is unclear and remains debatable (Verdoodt et al., 2018). Besides being highly incident, SIM is a major contributor to statin discontinuation (Ward et al., 2019). Therefore, our narrative review will focus on myotoxicity alone.

## Statin-Induced Myotoxicity

Statin-induced myotoxicity presents as fatigue, muscle pain, muscle tenderness, muscle weakness, nocturnal cramping, or tendon pain (Sathasivam and Lecky, 2008). A retrospective investigation of 45 patients with statin-associated myopathy showed that the time to onset of muscle symptoms was 6 months on average, whereas time to symptom resolution was reported to be approximately 2 months after statin discontinuation (Hansen et al., 2005). SIM is concentration-dependent (Hu et al., 2012). Because of the complex interplay between PK and pharmacodynamics (PD), a drug’s exposure is often defined by the dose administered and its effects (Wright et al., 2011; Sandritter et al., 2017). McClure et al. (2007) identified myositis cases from the United Kingdom General Practice Research Database between the year 1999 and 2003. Using a 40 mg mean daily dose equivalent as cutoff threshold, subjects taking statins were classified into high-dose (> 40 mg) and low-dose (< 40 mg) groups. Their analysis showed that a high mean daily statin monotherapy dose conferred a sixfold greater myositis risk (McClure et al., 2007). Supporting that, an observational study [Prediction of Muscular Risk in Observational Conditions (PRIMO)] conducted in 2005 on nearly 8,000 hyperlipidemic patients revealed that SIM in high statin-dosage (80 mg fluvastatin, 40 or 80 mg atorvastatin, 40 mg pravastatin, 40 or 80 mg simvastatin) therapy is greatly under-reported (Bruckert et al., 2005).

It can be challenging to design and report studies on SIM, mainly due to the heterogeneity and non-standardized classification of SIM, especially myalgia. Clinically, SIM can be assessed subjectively and objectively. The most common example for subjective and objective assessment is a patient’s symptoms and plasma creatine kinase (CK) concentrations, respectively. While both subjective and objective parameters are often applied in diagnosing severe SIM such as myopathy and rhabdomyolysis, milder forms of myalgia are commonly diagnosed using symptoms alone. Therefore, the different approaches used may account for the variable incidence rate of SIM, as well as non-replicated findings. Hence, various efforts have been undertaken by the academic field to propose suitable definitions for SIM (Thompson et al., 2016). In 2014, a six category statin-related myotoxicity (SRM) classification was proposed by the European Phenotype Standardization Project (Table 2), which included a rare and recently discovered type of SIM: anti-HMGCR myopathy. It is a subtype of autoimmune-mediated necrotizing myositis, which is characterized by severe proximal weakness, myofiber necrosis, and occasional extramuscular involvement (Pinal-Fernandez et al., 2018). In addition to phenotype grouping, a statin myalgia index score algorithm was proposed by the National Lipid Association (Rosenson et al., 2014).

**TABLE 2:** Phenotype classification of statin-related myotoxicity [adapted from Alfirevic et al. (2014)].

SRM classification	Phenotype	Definition
SRM 0	CK elevation < 4 × ULN	No muscle symptoms
SRM 1	Myalgia, tolerable	Muscle symptoms without CK elevation
SRM 2	Myalgia, intolerable	Muscle symptoms, CK < 4 × ULN, complete resolution on dechallenge
SRM 3	Myopathy	CK elevation > 4 × ULN < 10 × ULN ± muscle symptoms, complete resolution on dechallenge
SRM 4	Severe myopathy	CK elevation > 10 × ULN < 50 × ULN, muscle symptoms, complete resolution on dechallenge
SRM 5	Rhabdomyolysis	CK elevation > 10 × ULN with evidence of renal impairment + muscle symptoms or CK > 50 × ULN
SRM 6	Autoimmune-mediated necrotizing myositis	HMGCR antibodies, HMGCR expression in muscle biopsy, incomplete resolution on dechallenge

Despite the plethora of definitions of SIM, all agree that SIM can occur with or without CK elevation. Ranging from myalgia, myositis, myonecrosis, to rhabdomyolysis, it is understood that all of these symptoms represent a gradation along the same pathological pathway or pathways (Thompson et al., 2016). Unfortunately, the pathogenesis of SIM has not been clearly established, and this further complicates SIM categorization. To date, there are several mechanisms proposed, including the following:

1) A supply disruption of farnesyl and geranyl pyrophosphate, which results from statins blocking the downstream products of the mevalonate pathway (Jaskiewicz et al., 2018). These two end products are involved in maintaining cell growth and preventing apoptosis. This idea was proposed when squalene synthase inhibitor, which blocks cholesterol synthesis without affecting other end products of the mevalonate pathway, did not induce myotoxicity *in vitro* (Flint et al., 1997).
2) A localized impact on cholesterol biosynthesis. Statins have been shown to reduce cholesterol content in skeletal muscle cells (Nishimoto et al., 2003), which will disturb the stability of the cell membrane (Westwood et al., 2005). Because ions channels and transporters are embedded within the membrane, it was suggested that a change in the structure of skeletal cell membrane might disturb ion conductance, thus impairing muscle membrane excitability (Pierno et al., 1995).
3) Mitochondrial dysfunction resulting from coenzyme Q_10_ depletion (Marcoff and Thompson, 2007; Hargreaves, 2014) and calcium ion (Ca^2+^) leakage (Lotteau et al., 2019). The latter is a recent proposed pathway. It was shown that statin treatment dissociated FK506 binding protein from the ryanodine receptor 1 (RYR1), which is important for calcium regulation within the sarcoplasmic reticulum (SR) (Brillantes et al., 1994). This led to an excessive release of Ca^2+^ referred to as Ca^2+^ sparks (Lotteau et al., 2019). Impaired Ca^2+^ signaling pathway has been associated with several muscular disorders including myopathy and dystrophy (Agrawal et al., 2018). Because RYR1 is associated with proapoptotic signaling through reactive nitrogen or oxygen species, it was proposed that statin-mediated RNS/ROS-dependent destabilization of SR Ca^2+^ handling may initiate skeletal muscle toxicity (Lotteau et al., 2019).

While the lactone moiety of statins is suggested to induce SIM (Skottheim et al., 2008), it is important to note that some of the mechanisms were hypothesized based on the notion that statin lactones readily penetrate into muscle cells, facilitated by their lipophilicity (Taha et al., 2016). However, this does not explain SIM cases associated with the use of the hydrophilic statins (rosuvastatin and pravastatin) and simvastatin acid (Link et al., 2008). As discussed, SIM is a dose-dependent class effect of statins (Raju et al., 2013). Besides dose, there are other risk factors that have been identified to predispose a patient to develop SIM. These can be divided into genetic and non-genetic risk factors. Non-genetic factors include age 65 years or older (Schech et al., 2007), small body frame (Pasternak et al., 2002), Asian ethnicity (Birmingham et al., 2015), female gender (Puccetti et al., 2010), renal disease (Hedenmalm et al., 2010), and drug–drug interactions with concurrent administration of CYP3A4 inhibitors (Neuvonen, 2010). The focus of this review is genetic factors.

Improved understanding of relevant genetic factors may offer a strategy to predict the risk of SIM and allow improved targeting of statin therapy to the right patients. There is a wide range of genetic variation that is likely to be associated with the risk of developing SIM. This review aims to summarize the genetic evidence for SIM.

## Pharmacogenetics of Statin-Induced Myotoxicity

The study of how genes can impact drug disposition is commonly referred to as *pharmacogenetics*. This can refer to an individual gene, a therapeutic area, or an individual drug. Variation in a drug’s PK and PD is the focus of pharmacogenetics, which in turn can impact on the safety and efficacy profile of a drug (Daly, 2017). The majority of pharmacogenetic associations were discovered using candidate gene association studies (Feng et al., 2012) or genome-wide association studies (GWAS). For SIM, the first pharmacogenetic association was reported with a hepatocyte uptake transporter: solute carrier organic anion transporter family member 1B1 (*SLCO1B1*) gene in a 2008 GWAS (Link et al., 2008). Over the subsequent years, studies exploring other genes and their association with SIM have expanded significantly. A list of GWAS studies discussed in this review is as summarized in Table 7. This review will detail the association of genetic variation in enzymes and transporters involved in statin disposition.

## Pharmacokinetics

An important PK parameter in drug studies is the concentration of the drug’s moiety known for delivering either therapeutic response or resulting in adverse drug reactions (ADRs). Genetic variation in enzymes and transporters to be discussed here have been reported to influence the concentration of statins in the body, which in turn may increase the risk of developing SIM.

### Metabolic Enzymes

Statin-induced myotoxicity is known to be dose-related (Barry et al., 2018). Drug-metabolizing enzymes that affect statin PK by impacting upon oral bioavailability and clearance therefore can alter the risk of SIM. Since CYP450 enzymes were discovered 50 years ago, these enzymes, and their genes, have been the focus of a great deal of research (Estabrook, 2003). The associations between *CYP450* genetic variation and variable PK parameters of SIM are discussed below.

#### Cytochrome P450 2D6

**FIGURE 3:**
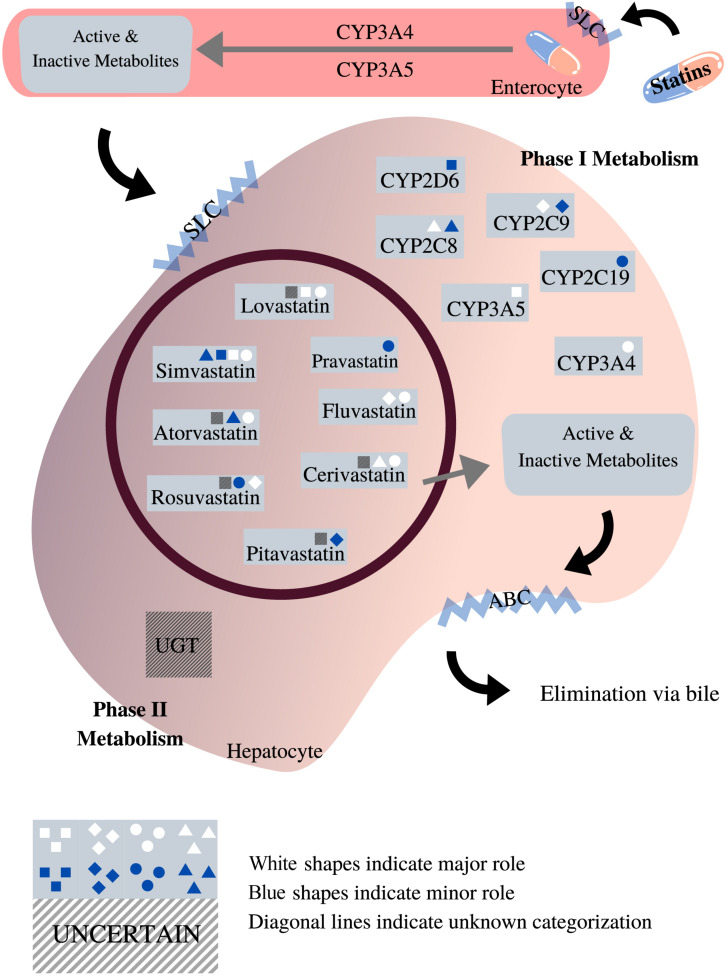
Roles of phases I and II enzymes in statin metabolism [adapted from Goldberg and Roth (1996), Bischoff et al. (1997), Hu et al. (2009), Sirtori et al. (2012), Whirl-Carrillo et al. (2012), Maji et al. (2013), Schirris et al. (2015)]. This diagram depicts the pharmacokinetic pathway of statins, facilitated by endogenous transporters, e.g., SLC and ABC transporters. All statins undergo both phases I and II metabolism prior to elimination. As shown, the impact exerted by different phase I metabolic enzymes on statins varies according to the type of statin. Unlike phase I, the role of phase II metabolic enzymes in statin metabolism is unclear, designed with Canva.com.

The *CYP2D6* gene is the most polymorphic among the *CYP450* genes, with more than 100 different alleles that have been identified to date (Nofziger et al., 2020). Many of these variants influence CYP2D6 enzyme function and have been associated with toxicity and altered efficacy for a variety of drugs. The CYP2D6 enzyme metabolizes up to 25% of commonly prescribed medications. However, the role of CYP2D6 in statin metabolism is minor (Figure 3), and research that has investigated the association between genetic variation in *CYP2D6* and SIM has reported mixed conclusions (Table 3).

**TABLE 3:** Summary of CYP2D6 studies.

Study design	Sample size	Ethnicity	*SNPs*	Statins	Findings	Reference
Case–control	Case: 50 Control: 50	Italian	*1 (wild type), *4 (rs3892097)	Simvastatin Fluvastatin	Efficacy, with respect to serum cholesterol concentration was twofold greater in study participants with *4/*4 genotype.	(Zuccaro et al., 2007)
Randomized open-label	452	Caucasian, African American and others	*4 (rs3892097), *10 (rs1065852, rs1135840)	Atorvastatin Pravastatin Simvastatin	No correlation between *4 and *10 carriers with adverse effects.	(Voora et al., 2009)
Single-dose pharmacokinetic	23	Chinese	*1 (wild type), *2 (rs16947, rs1135840) *5 (deletion), *10 (rs1065852, rs1135840)	Lovastatin	A gene–dose effect was observed (*p* < 0.01). When compared to *1 or *2, the mean area under the curve (AUC) of lovastatin lactone increased at a ratio of 1.57, 2.11, 2.52, and 5.84 in *1 or *2/*10, *10/*10, *5/*10, and *10/*10, respectively.	(Yin et al., 2012)
Retrospective cohort	88	Not reported	*2xN (duplication), *3 (rs35742686), *4 (rs3892097), *5 (deletion)	Simvastatin	Increasing intolerability was reported with increasing number of alleles. 17% in subjects with no defective alleles, 46% in subjects with one defective allele, and 80% in subjects with two defective alleles.	(Mulder et al., 2001)
Case–control	Case: 75 Control: 188	European, sub-Saharan, east Asian and Indigenous American	*4 (rs3892097)	Atorvastatin Simvastatin	Carriers of the *4 allele were twice as likely to suffer from atorvastatin and simvastatin-induced muscle toxicity (*p* < 0.001, OR = 2.2).	(Frudakis et al., 2007)
Single-dose randomized open label	133	Korean	*10 (rs1065852, rs1135840), *5 (deletion), *14 (rs16947),*41 (rs28371725)	Simvastatin	Significantly higher AUC of simvastatin lactone in study participants with the *5 allele (*p* = 0.026) and *14 allele (*p* = 0.015).	(Choi et al., 2015)

Evidence for the role of CYP2D6 in metabolizing statins was initially published by Nordin et al. (1997) where they reported a negative correlation between simvastatin-treated serum cholesterol and the debrisoquine metabolic ratio (ratio of urinary debrisoquine to its hydroxy metabolite). However, this research assessed only 10 samples of simvastatin. With respect to SIM, Frudakis et al. (2007) reported a significant (*p* = 0.001) association between atorvastatin treatment and SIM in samples with *CYP2D6^∗^4* polymorphism.

As shown in Table 3, results reported by literature exploring the association of *CYP2D6* single-nucleotide polymorphisms (SNPs) with SIM are mixed. It is more likely that CYP2D6 in combination with other metabolic pathways discussed below may account for the variation in response and muscle toxicity reported by statin users.

#### Cytochrome P450 2C9

The CYP2C family consists of CYP2C8, CYP2C9, CYP2C18, and CYP2C19 (Daly et al., 2017). CYP2C9 and CYP2C19 are responsible for metabolizing up to 20% of clinical drugs (Hirota et al., 2013). CYP2C9 is the main enzyme involved in the metabolism of fluvastatin (Fischer et al., 1999) and rosuvastatin (Martin et al., 2003; Luvai et al., 2012) and has a minor role in the metabolism of pitavastatin (Catapano, 2010). The *CYP2C9* gene has more than 60 reported genetic variants (Gaedigk et al., 2018), of which two commonly studied *CYP2C9* genotypes are the missense variants *CYP2C9^∗^2* (rs1799853) and *CYP2C9^∗^3* (rs1057910). *CYP2C9* genetic variations have been reported to influence the PK profile of fluvastatin and pitavastatin. In an analysis of 12 healthy Chinese participants, *^∗^3* heterozygotes were shown to have a significantly higher area under the concentration–time curve (AUC) of pitavastatin acid (*p* = 0.000) and pitavastatin lactone (*p* = 0.001) (Zhou et al., 2013a). With respect to fluvastatin, Kirchheiner et al. (2003) recruited 24 healthy volunteers and the cohort received a 40 mg racemic dose of fluvastatin daily for 2 weeks. The measured AUC of 3S,5R-fluvastatin enantiomer increased in a gene-dose–dependent manner from non-carriers (227μg/L), *^∗^3* heterozygotes (360μg/L) to *^∗^3* homozygotes (1126μg/L) (Kirchheiner et al., 2003). In contrast, a PK study that assessed the impact of *CYP2C9* genetic variation in 162 study participants reported no association (DeGorter et al., 2013). However, *^∗^3* allele carriers were shown to have a significantly (*p* < 0.01) lower plasma level for one of rosuvastatin’s metabolites, *N*-desmethyl rosuvastatin (Bai et al., 2019).

There are limited studies investigating the association between SIM (Frudakis et al., 2007; Zuccaro et al., 2007) and genetic variability in *CYP2C9*. With respect to ADRs, a study of 104 Croatian patients reported a 2.5-fold higher risk for homozygotes and heterozygotes of *^∗^2* and *^∗^3* alleles in developing ADRs (defined by the study as myotoxicity and hepatotoxicity) (*p* = 0.037) (Mirosevic Skvrce et al., 2013).

Therefore, *CYP2C9* genetic variation may be a risk factor impacting the metabolism of fluvastatin, rosuvastatin and pitavastatin.

#### Cytochrome P450 2C19

*CYP2C19* is the next most polymorphic CYP2C family member gene after *CYP2C9*. It has more than 30 star alleles in the Pharmvar database (Gaedigk et al., 2019). The two most commonly studied genetic variants that lead to reduced CYP2C19 function are *CYP2C19^∗^2* (rs4244285) and *CYP2C19^∗^3* (rs4986893). Another extensively researched variant is *CYP2C19^∗^17* (rs12248560), which is known to increase the enzymatic activity of CYP2C19 (Scott et al., 2012).

CYP2C19 is reported to play a minor role in the metabolism of rosuvastatin (Bellosta et al., 2004). A PK study in 49 healthy Taiwanese participants investigated the impact of *CYP2C19^∗^2* and *^∗^3* alleles on rosuvastatin and its metabolites. There were no reported differences in the AUC of rosuvastatin, rosuvastatin lactone, and *N*-desmethyl rosuvastatin between normal metabolizers of statins and participants with null function CYP2C19 (Finkelman et al., 2015). This result was supported by Lee et al. (2013), who also reported no effect of *CYP2C19* variation on rosuvastatin metabolites in a Chinese cohort (Lee et al., 2013). While other statins are not known to be specific substrates of CYP2C19, studies have explored the association of *CYP2C19* genotypes with PK and the safety profile of simvastatin and atorvastatin. Choi et al. (2015) showed that the AUC of simvastatin lactone increased significantly with the number of *CYP2C19^∗^2* alleles (*p* = 0.022). However, no effects were observed with simvastatin acid. In addition, a further study reported that both the *CYP2C19^∗^2* and *^∗^3* alleles were not associated with SIM induced by simvastatin or atorvastatin (Frudakis et al., 2007).

#### Cytochrome P450 2C8

The CYP2C8 enzyme is responsible for metabolizing an estimated 5% of prescribed medications such as montelukast, paclitaxel, and rosiglitazone (Naraharisetti et al., 2010; Hanioka et al., 2010; Karonen et al., 2012; Bazargan et al., 2017). To date, CYP2C8 is a known metabolic enzyme for one statin only—cerivastatin, which was withdrawn from the market in 2001 and is no longer in clinical use (Furberg and Pitt, 2001). Cerivastatin when coprescribed with a strong CYP2C8 inhibitor such as gemfibrozil significantly increased the risk of rhabdomyolysis (Kaspera et al., 2010). For this reason, cerivastatin was withdrawn from the market (Furberg and Pitt, 2001). Like other CYPs discussed, CYP2C8 is likely to play a minor role in the metabolism of other statins. With respect to *CYP2C8* variation resulting in SIM, two studies identified showed no significant correlation between *CYP2C8* SNPs (*^∗^2*, *^∗^3*, and *^∗^4*) and SIM (Voora et al., 2009; Marciante et al., 2011).

#### Cytochrome P450 3A4/3A5

The CYP3A subfamily is the largest human liver cytochrome enzyme system with a 29-fold variability in expression between individuals (Michaels and Wang, 2014). The CYP3A enzyme family comprises CYP3A4 and CYP3A5, which are the main metabolic pathways for most statins (Wang et al., 1991; Prueksaritanont et al., 1997, 2003; Igel et al., 2001; Neuvonen et al., 2008; Rowan et al., 2012). Simvastatin is reported to be preferentially metabolized by CYP3A4 (Prueksaritanont et al., 1997).

Simvastatin and lovastatin are administered as lactone prodrugs and require bioactivation by esterases through hydrolysis (Igel et al., 2001; Egom and Hafeez, 2016) before being oxidized by CYP3A4 into active metabolites (Huttunen et al., 2011). While other statins do not require bioactivation, CYP3A4 is the main enzyme involved in their metabolism (Liu et al., 2019). Administration of CYP3A4 inhibitors such as itraconazole concurrently with statins results in a 2.5-fold increase in the plasma concentration of atorvastatin (Mazzu et al., 2000). Similarly, concurrent administration of cimetidine and lovastatin reduced the formation of 6-hydroxy-lovastatin by 20% (Vyas et al., 1990b). Ciclosporin, a potent CYP3A4 inhibitor, is known to interact with most statins. Studies have reported that statins coadministered with ciclosporin resulted in an approximately 2-, 3-, 6-, and 20-fold increase in AUC for fluvastatin (Goldberg and Roth, 1996), simvastatin (Arnadottir et al., 1993), atorvastatin (Asberg et al., 2001), and lovastatin (Olbricht et al., 1997) respectively. In addition, ciclosporin is reported to impact other pathways important in statin disposition such as inhibiting SLC transporters (Kalliokoski and Niemi, 2009).

For CYP3A5, the most commonly studied polymorphism is *CYP3A5^∗^3* (rs776746), which produces non-functional CYP3A5 enzyme (Kuehl et al., 2001; Lamba et al., 2012). Therefore, for individuals who express CYP3A5, the metabolism of substrate medication may be enhanced (Passey et al., 2011; Lamba et al., 2012). Shin et al. (2011) found that the AUC of atorvastatin lactone was 36% higher in non-expressors (*CYP3A5^∗^3*) than expressors (*CYP3A5^∗^1*) (*p* = 0.038). However, this was not replicated by Hermann et al. (2006), who reported a significant (*p* < 0.01) 2.4-fold higher AUC level of atorvastatin lactone in SIM cases but did not find any association with *CYP3A5*.

Unlike other CYPs, genes from the CYP3A family are not highly polymorphic (Wang and Sadee, 2012). This may also account for the inconsistent findings for association studies of genetic variation for both *CYP3A4* and *CYP3A5* with SIM. These studies are summarized in Table 4.

**TABLE 4:** Summary of CYP3A studies.

Study design	Sample size	Ethnicity	*Gene or SNPs*	Statins	Findings	Reference
Randomized open-label	452	Caucasian, African American, and others	CY3A4*1b (rs2740574)	Atorvastatin Pravastatin Simvastatin	No correlation between *1b alleles with either discontinuation of therapy, myalgia or creatinine kinase elevation of 3 × or more from the upper limit normal.	(Voora et al., 2009)
Prospective Cohort	1198	Caucasian	CY3A4*1b (rs2740574)	Atorvastatin Simvastatin	Change or reduction of dose is two times lower in *1b carriers (*p* = 0.023). Incidental findings: no correlation between *1b with pravastatin.	(Becker et al., 2010)
Single-dose pharmacokinetic	44	Chinese	CYP3A4*1G (rs2242480)	Lovastatin	No association between *1G allele and AUC of lovastatin acid or lovastatin lactone.	(Zhao et al., 2017)
Case–control	Case: 75 Control: 188	European, sub-Saharan, East Asian, and indigenous American	CYP3A5*3 (rs776746)	Atorvastatin Simvastatin	No correlation of *3 allele with SIM.	(Frudakis et al., 2007)
Case–control	Case: 50 Control: 50	Not reported	CYP3A5*3 (rs776746)	Atorvastatin Simvastatin	No significant association of *3 allele with SIM.	(Zuccaro et al., 2007)
Single-dose pharmacokinetic	22	Korean	CYP3A5*3 (rs776746)	Simvastatin	Significant increasing trend observed for AUC of simvastatin across *1/*1, *1/*3, and *3/*3 (*p* = 0.013).	(Kim et al., 2007)
Single-dose randomized open label	133	Korean	CYP3A5*3 (rs776746)	Simvastatin	No significant effects of *3 on AUC of simvastatin acid or simvastatin lactone.	(Choi et al., 2015)
Case–control	Case: 38 Control: 164	South Indian	CYP3A5*3 (rs776746)	Atorvastatin Rosuvastatin	Non–statin-specific analysis showed an OR of 2.14 for subjects with CYP3A5*3 CC genotype in developing myopathy (*p* = 0.04). No significant effects observed in statin-specific analysis for both atorvastatin and rosuvastatin.	(Ramakumari et al., 2018)
Single-dose pharmacokinetic	12	Chinese	CYP3A5*3 (rs776746)	Pitavastatin	No significant effects of *3 on AUC of pitavastatin acid or pitavastatin lactone.	(Zhou et al., 2013a)
Prospective cohort	830	Caucasian and African American	CYP3A5*3 (rs776746) CYP3A4*22 (rs35599367)	Simvastatin	Significantly higher plasma concentration of simvastatin acid (14%) (*p* = 0.04) and simvastatin lactone (20%) (*p* = 0.06) in non-Finnish Europeans with CYP3A4*22 allele. Significantly higher plasma concentrations of simvastatin lactone (170%) (*p* < 0.01) for African Americans with CYP3A4*22 allele. No association with simvastatin acid. No association between plasma concentration of both simvastatin acid and simvastatin lactone in CYP3A5*3 carriers in non-Finnish Europeans. Significantly higher plasma concentrations of simvastatin lactone (33%) (*p* = 0.02) for non-expressors (*3/*3) in African Americans. No association with simvastatin acid.	(Kitzmiller et al., 2014)
Retrospective cohort	299	Caucasian	CYP3A5*3 (rs776746) CYP3A4*22 (rs35599367)	Atorvastatin	No association with atorvastatin concentration.	(DeGorter et al., 2013)
Prospective cohort	116	European	CY3A4*1b (rs2740574) CYP3A5*3 (rs776746)	Simvastatin	No association with lipid and lipoprotein levels (efficacy), and myalgia (tolerability).	(Fiegenbaum et al., 2005)
Case–control	Case: 68 Control: 69	Caucasian	CY3A4*1b (rs2740574) CYP3A5*3 (rs776746)	Atorvastatin	Serum creatine kinase (CK) level was 25% greater in CYP3A5*3 homozygotes when compared to heterozygotes (*p* = 0.025 without concomitant gemfibrozil and *p* = 0.01 without concomitant gemfibrozil and niacin). No significant association between CYP3A4*1B with serum CK level.	(Wilke et al., 2005)

#### Uridine 5′-Diphosphoglucuronosyltransferase

The UGT is an important enzyme in phase II metabolism. It conjugates xenobiotics or endogenous compounds with glucuronic acid to facilitate their excretion from the body including the lactonization of statins (Schirris et al., 2015; Sanchez-Dominguez et al., 2018). An estimated 10% of administered simvastatin hydroxy acid undergoes lactonization through glucuronidation (Prueksaritanont et al., 2002). This superfamily consists of three subfamilies, UGT1A, UGT2A, and UGT2B (Mackenzie et al., 2005). The *UGT1A* gene undergoes alternative splicing resulting in the transcription of nine functional isoforms and four pseudogenes (Gong et al., 2001). Among the isoforms, *UGT1A1* is the most commonly studied gene associated with drug toxicity (Liu et al., 2014) and disease (Servedio et al., 2005). While there are more than 100 *UGT1A1* variants recorded (Barbarino et al., 2014), to date, only *UGT1A1^∗^28* (rs3064744) and *UGT1A^∗^80* (rs887829) have been associated with PK variability and toxicity of statins. *UGT1A1^∗^28* is characterized by the presence of seven TA dinucleotide repeats, instead of the normal six repeats, in the promoter region, which results in decreased UGT1A1 expression (Sanchez-Dominguez et al., 2018). *UGT1A^∗^80* is a SNP located within 300 base pairs of the *UGT1A1^∗^28* TA repeat and in strong linkage disequilibrium with it (Gammal et al., 2016).

As mentioned earlier, the lactone forms of statins are reported to induce SIM (Skottheim et al., 2008). Several studies have investigated the association of *UGT1A1* variants and their effect on PK parameters of statin lactones. Stormo et al. (2013) reported a significant reduction in atorvastatin lactone plasma concentrations in *UGT1A1^∗^28* carriers when compared with non-carriers (*p* < 0.05). This observation was in contrast with Riedmaier et al. (2010), who showed a higher atorvastatin lactone plasma concentration in individuals with the *UGT1A1^∗^28* polymorphism. Riedmaier et al. (2010) did both *in vitro* and *in vivo* tests on an estimated 150 human liver microsome samples and 56 Caucasian volunteers, respectively. The genetic analysis conducted *in vitro* reported a significant gene–dose effect between *UGT1A1^∗^28* allele and the plasma concentration of atorvastatin lactone (*p* < 0.01). A recent GWAS investigated the levels of atorvastatin and its metabolites, 2-hydroxyatorvastatin, 2-hydroxyatorvastatin lactone, and atorvastatin lactone, in patients taking either 40 or 80 mg of atorvastatin. *UGT1A1^∗^80* was significantly associated (*p* = 7.25 × 10^–16^) with a higher metabolic ratio of 2-hydroxyatorvastatin/atorvastatin and 2-hydroxyatorvastatin lactone/atorvastatin lactone (*p* = 3.95 × 10^–15^) (Turner et al., 2020).

UGT1A3, another UGT isoform, has also been correlated with the lactonization of atorvastatin. In a study with 24 Korean participants, an increasing trend was observed for the mean atorvastatin lactone AUC across carriers of the *UGT1A3^∗^2* allele (Cho et al., 2012). However, this observation was not supported by Riedmaier et al. (2010). The *in vivo* test conducted by Riedmaier et al. (2010) showed that only the AUC of atorvastatin metabolite, 2-hydroxyatorvastatin lactone, was significantly (*p* < 0.05) associated with *UGT1A3^∗^2* allele. No significance was observed with either atorvastatin or atorvastatin lactone (Riedmaier et al., 2010).

### Transporters

Endogenous transporters play an important role in the PK of drugs and xenobiotics by controlling the influx of nutrients and efflux of waste material (Mao et al., 2018). ABC and SLC are two major families of transporters (Giacomini et al., 2010), where variations in the genes of these transporters have been shown to influence the disposition of statins and risk of SIM (Niemi, 2010).

#### Solute Carrier Organic Anion Transporter Family Member 1B1/Organic Anion Transporter Polypeptide-1B1

Previously known as *organic anion transporter polypeptide 1B1* (*OATP1B1*), the *SLCO1B1* gene encodes for a transporter located along the basolateral membrane of hepatocytes (Lee and Ho, 2017). This transporter mediates the hepatocellular intake of endogenous molecules and drugs such as statins (Oshiro et al., 2010). Statins exert their lipid-lowering mechanism and undergo metabolism, within hepatocytes (Stancu and Sima, 2001; Whirl-Carrillo et al., 2012). Therefore, *SLCO1B1* polymorphisms are expected to impact upon statin disposition and contribute to SIM.

An association between the genetic variation of *SLCO1B1* gene and SIM was initially discovered by a GWAS comprising of 85 participants with suspected simvastatin-myopathy and 90 matched controls. A significant (*p* = 4 × 10^–9^) association was found with the rs4363657 intronic SNP with an odds ratio (OR) of 4.3 per variant allele and 17.4 for both alleles. The rs4363657 (*SLCO1B1*) is in almost complete linkage disequilibrium with a missense variant, rs4149056. This SNP (rs4149056) was significantly associated (*p* < 2 × 10^9^) with SIM, with an OR of 4.5 and 16.9 in heterozygotes and homozygotes, respectively. In a subsequent replication study conducted on 21 participants from the Heart Protection Study, rs4149056 association with SIM was found to be significant (*p* = 0.004). In addition, further SNPs discovered via the GWAS (rs2306283, rs11045819, and rs34671512) were reported to have marginal to no increased risk of SIM (Link et al., 2008).

Since then, the two most commonly studied *SLCO1B1* SNPs associated with SIM have been rs4149056 and rs2306283. The *SLCO1B1* alleles defined by these SNPs are *^∗^5* (rs4149056) and *^∗^1b* (rs2306283), whereas individuals who carry both the alleles are assigned the *^∗^15* haplotype (Nozawa et al., 2002). The reference allele (wild type) for *SLCO1B1* is defined as *^∗^1a* with normal transporter function (Ramsey et al., 2014). *SLCO1B1 ^∗^1b* is reported to have functional activity parallel to *^∗^1a* (Tirona et al., 2001; Nozawa et al., 2002), although substrate-dependent activity has been reported for pravastatin (Mwinyi et al., 2004; Maeda et al., 2006). Meanwhile, *SLCO1B1 ^∗^5* is associated with reduced transporter function (Tirona et al., 2001). The *^∗^15* haplotype, initially discovered in a Japanese population, is reported to be a decreased function variant (Nozawa et al., 2002; Ramsey et al., 2014). A summary of commonly studied *SLCO1B1* SNPs is listed in Table 5.

**TABLE 5:** Common SNPs of the SLCO1B1 gene associated with SIM [adapted from Ramsey et al. (2014)].

Phenotype	Genotype/ haplotype involved	Identified diplotype	Diplotype definition	Genotype at rs4149056
Normal function	*1a (wild type) *1b (rs2306283)	*1a/*1a, *1a/*1b, *1b/*1b	Two normal function alleles	TT
Intermediate function	*1a (wild type) *1b (rs2306283) *5 (rs4149056) *15 (rs2306283, rs4149056) *17 (rs2306283, rs4149056, rs4149015)	*1a/*5, *1a/*15, *1a/*17, *1b/*5, *1b/*15, *1b/*17	One normal function allele and one reduced function allele	TC
Low function	*5 (rs4149056) *15 (rs2306283, rs4149056) *17 (rs2306283, rs4149056, rs4149015)	*5/*5, *5/*15, *5/*17, *15/*15/, *15/*17, *17/*17	Two reduced function alleles	CC

To elucidate the pharmacogenetic association of SLCO1B1 transporters and SIM, it is crucial to understand the PK relationship between statins and *SLCO1B1* genotype. Mwinyi et al. (2004) conducted a PK study of pravastatin in 30 healthy male subjects. The AUC in participants homozygous for *^∗^1a* and *^∗^1b* did not show any significant differences. However, the AUC in individuals carrying the *^∗^5* allele was twofold higher than *^∗^1b* (*p* = 0.002) and *^∗^1a* (*p* = 0.049) (Mwinyi et al., 2004). Similarly, several studies have reported no significant difference between wild type and *^∗^1b* homozygotes in the AUC measured for rosuvastatin (Choi et al., 2008; Lee et al., 2013; Liu et al., 2016) and simvastatin acid (Choi et al., 2015). However, when *^∗^1b* was analyzed in 299 participants taking atorvastatin or rosuvastatin, a lower atorvastatin plasma concentration was reported (*p* < 0.01) in carriers of *^∗^1b* (DeGorter et al., 2013). Pasanen et al. (2006) genotyped 32 young healthy Caucasians after a single dose of 40 mg simvastatin and showed that the peak plasma concentration of active simvastatin acid in *^∗^5* homozygotes was 162 and 200% higher when compared with *^∗^5* heterozygotes and non-*^∗^5* carriers. In addition, further studies have supported the association of significantly elevated simvastatin acid AUC with the *SLCO1B1^∗^5* allele (Choi et al., 2008; Voora et al., 2009; Jiang et al., 2017).

Furthermore, de Keyser et al. (2014) analyzed the *SLCO1B1* genotype from an estimated 1,900 subjects and found that those currently prescribed simvastatin and also carriers of two *^∗^5* alleles had a 1.7-fold higher risk of statin dose reduction or change of cholesterol lowering drug due to statin-associated ADRs (*p* = 0.0033). When compared with wild type, *^∗^5* homozygotes were observed to achieve 144, 100, and 65% greater mean AUC for atorvastatin, 2 hydroxy-atorvastatin, and rosuvastatin respectively (Pasanen et al., 2007).

As discussed earlier, the GWAS conducted by Turner et al. (2020) also reported a significant association between the *^∗^5* allele and the level of atorvastatin (*p* = 2.21 × 10^–6^) and its metabolite, 2-hydroxy atorvastatin (*p* = 1.09 × 10^–6^). The observed significant association between *^∗^5* alleles and the plasma concentration of statin was supported by other studies on pravastatin (*p* = 0.01) (Ho et al., 2007), rosuvastatin (*p* = 0.019) (Lee et al., 2013), atorvastatin (*p* < 0.05) (DeGorter et al., 2013), and the AUC of both pitavastatin acid (*p* = 0.00) and pitavastatin lactone (*p* = 0.007) (Zhou et al., 2013a). Birmingham et al. (2015) reported that the average AUC of atorvastatin, rosuvastatin, and simvastatin acid was higher in *SLCO1B1 ^∗^5* allele carriers. This trend was similar across all three ethnic groups (Chinese, Japanese, and European). Meanwhile, no effect was reported with simvastatin lactone.

Because both *^∗^5* and *^∗^15* result in impaired SLCO1B1 transporter function, the PK impact of *^∗^15* on statins is expected to be similar to that of *^∗^5*. Choi et al. (2008) found that Korean carriers of *^∗^15* had a twofold higher AUC of rosuvastatin compared with *^∗^1a* and *^∗^1b* homozygotes. This observation was supported by a recent study in a Chinese population (*p* = 0.015) (Liu et al., 2016). For pravastatin and pitavastatin, carriers of the *^∗^15* haplotype had a twofold and threefold higher AUC when compared with *^∗^1a*, respectively (Deng et al., 2008). Other PK studies on either pitavastatin (Oh et al., 2013) or pravastatin (Niemi et al., 2004; Ho et al., 2007) also reported significant associations with the *^∗^15* allele. With respect to atorvastatin and lovastatin, significantly higher AUCs of atorvastatin acid (*p* = 0.0018), 2-hydroxyatorvastatin acid (*p* = 0.0123), and lovastatin acid (*p* < 0.005, *p* = 0.072) were associated with the *^∗^5* or *^∗^15* alleles (Tornio et al., 2015). No significant association was found for the lactone forms of both atorvastatin and lovastatin (Lee et al., 2010; Tornio et al., 2015; Zhao et al., 2017).

A recent study conducted with Korean patients investigated the impact of *SLCO1B1* genetic variation on rosuvastatin according to phenotypes (Table 5), demonstrating that the mean plasma concentration of rosuvastatin showed an increasing trend across groups of normal function, intermediate function to low function (Kim et al., 2019).

##### Solute carrier organic anion transporter family member 1B1 polymorphisms and statin-induced myotoxicity

A recent GWAS that aimed to validate genetic risk factors for statin-induced myopathy in 128 myopathy cases successfully replicated the prior association of the *SLCO1B1^∗^5* allele with SIM (Carr et al., 2019). This SNP was significantly associated with statin-induced myopathy both in the initial case–control discovery cohort (*p* = 2.5 × 10^–9^), the replication study (*p* = 0.001), simvastatin cohort validation (*p* = 1.3 × 10^–11^), and final meta-analysis (*p* = 2.63 × 10^–18^). The replication cohort consisted of 19 myopathy cases and 585 statin-tolerant controls. The OR of SIM reported from this replication study was 3.98 for *SLCO1B1^∗^5* allele. Meanwhile, the meta-analysis combined the discovery and replication cohorts, resulting in a comparison of 271 myopathy cases with 7,493 controls. The OR of statin-associated SIM with the *^∗^5* allele was 2.99 in the meta-analysis (Carr et al., 2019). This finding has been supported by other studies, which reported an increased risk of SIM in *^∗^5* allele carriers. One study compared 12 cases and 39 controls and reported a threefold increased risk of myopathy in carriers of the *^∗^5* allele (*p* = 0.032) (Brunham et al., 2012). Meanwhile, Carr et al. (2013) showed that *^∗^5* heterozygote carriers have an OR of 2.13 for the risk of myopathy (*p* = 0.014). In addition, a recent GWAS on the circulating levels of atorvastatin and its metabolites reported *SLCO1B1 ^∗^5* allele was significantly associated with increased muscular symptoms (*p* = 0.016) and atorvastatin intolerance (*p* = 0.014) at an OR of 4 and 1.5, respectively (Turner et al., 2020).

A study that investigated *^∗^1b*, *^∗^5*, and *^∗^15* found that the presence of the *^∗^5* allele was associated with statin intolerance. In this study, intolerance was defined as any biochemical abnormality (CK or alanine aminotransferase levels) or prescribing change (statin switch or dose change) recorded. This study showed that the *^∗^5* allele was significantly (*p* = 0.02) associated with statin intolerance with an OR of 1.14 (Donnelly et al., 2011). In addition, a recent case–control study in a cohort of 606 Europeans reported that of 12 SNPs in nine candidate genes, only the *^∗^5* allele was significantly associated with SIM (OR = 1.73, *p* = 0.01) (Bakar et al., 2018).

There are several meta-analyses assessing the association of *SLCO1B1^∗^5* with SIM. The majority of them reported a significant association between the *^∗^5* allele and increased risk of SIM with an OR ranging from 1.57 to 2.09 (Hou et al., 2015; Lee and Chun, 2018; Xiang et al., 2018). Interestingly, another meta-analysis that investigated 13 studies showed that *SLCO1B1 ^∗^5* allele was associated with overall statin-induced adverse reactions, including SIM (Jiang et al., 2016). Furthermore, a meta-analysis on simvastatin specifically with 1,360 cases and 3,082 controls showed that *^∗^5* allele carriers had a threefold higher risk of SIM compared with non-carriers (Hou et al., 2015). Similarly, Xiang et al. (2018) with a larger number of cases (3265) and controls (7743) reported an OR of 2.35 for SIM in individuals receiving simvastatin.

Simvastatin is the most investigated statin with respect to SIM. However, other statins have been reported to significantly induce SIM. An observational case–control study in 76 European cases of muscular intolerance reported a threefold (OR = 2.7) risk for developing SIM with atorvastatin (*p* < 0.001) in individuals carriers of the *^∗^5* allele (Puccetti et al., 2010). For rosuvastatin, a significant association (*p* = 0.007) with SIM was observed in a Chinese population study consisting of 148 myotoxicity cases and 255 controls (Liu et al., 2017). In addition, Bai et al. (2019) reported a significant (*p* = 0.0052) association between *^∗^5* alleles and rosuvastatin-induced myopathy in an observational study of 758 Chinese patients with coronary artery disease. There is limited evidence with respect to *SLCO1B1^∗^5* and SIM in patients prescribed fluvastatin or pravastatin (Liu et al., 2017).

However, there have also been inconsistent findings reported for both SIM and PK variability with *SLCO1B1* polymorphisms. A PK study conducted with atorvastatin-related myopathy cases found that elevated AUCs of atorvastatin lactone and *p*-hydroxyatorvastatin lactone among patients suffering from atorvastatin-related myopathy were not associated with *SLCO1B1* polymorphisms or CK levels (Hermann et al., 2006). In addition, further studies have reported no significant association between *SLCO1B1^∗^5* and SIM for simvastatin (Khine et al., 2016; Liu et al., 2017), atorvastatin (Brunham et al., 2012; Carr et al., 2013; Hou et al., 2015; Liu et al., 2017; Ramakumari et al., 2018), and rosuvastatin (Puccetti et al., 2010; Danik et al., 2013; Ramakumari et al., 2018). Hubáček et al. (2015a) investigated the SNP rs4363657, which is in linkage disequilibrium with *^∗^5*, and they too, did not find an association with SIM. Two possible factors for the discordant results may be ethnic-dependent variation (Lee and Ho, 2017) and SLCO1B1 transporter substrate specificity (Niemi et al., 2011).

Ethnicity plays a major role in genetic variation. With regard to *SLCO1B1* polymorphisms, the prevalence of *^∗^5* is lower in African Americans or individuals of African descent (Ho et al., 2007; Santos et al., 2011; Khine et al., 2016), but higher in American Indians, where the genotype frequency observed is doubled (Santos et al., 2011). Interestingly, the association of SIM differs between ethnicities despite having similar allele frequencies. Although the genome aggregation database (gnomAD) reports the allele frequency of *SLCO1B1^∗^5* (rs4149056) in both non-Finnish Europeans and East Asians to be similar (∼15%), the significant association between rosuvastatin and SIM reported by Liu et al. (2017) among Chinese *^∗^5* allele carriers was not observed in individuals of European ancestry (Puccetti et al., 2010; Danik et al., 2013). However, other factors (study design, adequate sample size, and also the unexplainable higher plasma exposure of rosuvastatin observed in Asians when compared to non-Finnish Europeans) should be considered during interpreting ethnicity-driven differences (Lee et al., 2005). Another potential factor is the physical properties of different statins. Hydrophilic statins require functional SLC transporters in order to move across membranes or into cells. In contrast, lipophilic statins should be able to diffuse through the lipid membrane into extrahepatic tissues such as muscle, possibly increasing the risk of SIM (Bruckert et al., 2005; Neuvonen et al., 2006). This hypothesis is supported by PK studies reporting the impact of *SLCO1B1* polymorphisms on the acid form of simvastatin and rosuvastatin but not their lipophilic lactone structures (Pasanen et al., 2006; Choi et al., 2008).

A further factor that has been associated with *SLCO1B1* polymorphisms and SIM is an individual’s vitamin D status. Studies have reported an association between serum levels of vitamin D and SIM (Ahmed et al., 2009; Morioka et al., 2015; Glueck et al., 2017). Linde et al. (2010) genotyped a cohort of 46 patients on statin treatment and measured 25-hydroxyvitamin D levels. This analysis showed that carriers of the *^∗^5* allele had a threefold higher risk (*p* = 0.07) of developing myalgia regardless of their vitamin D status (sufficient or deficient). In contrast, Alghalyini et al. (2018) showed that the *^∗^5* allele increased the risk of SIM in vitamin D deficient subjects only (*p* = 0.022). Despite the inconsistency, these studies show the detrimental effect of the *^∗^5* allele.

From this review, it is evident that there is a strong association between *SLCO1B1* rs4149056 and SIM. The Clinical Pharmacogenetics Implementation Consortium (CPIC) has conducted a review on the available evidence and published a peer-reviewed guideline to assist in prescribing simvastatin in individuals who carry a higher risk of developing SIM (Ramsey et al., 2014). The clinical implementation of *SLCO1B1* testing is discussed later in this review.

#### Other Solute Carrier Transporters

SLCO1B3 and SLCO2B1 are two other members of the SLC transporter family. Similar to SLCO1B1, both SLCO1B3 and SLCO2B1 can be transporters for statins (Grube et al., 2006; Alam et al., 2018). However, their roles in statin disposition are still unclear because of the poor classification of genetic variation in these transporter genes. A PK study on rosuvastatin plasma concentration found no association with *SLCO1B3* (rs7311358) and *SLCO2B1* (rs12422149) (DeGorter et al., 2013). Furthermore, a study in 758 Chinese participants reported that *SLCO1B3* (rs7311358) was not significantly associated with variation in the plasma concentration of rosuvastatin and its two metabolites (Bai et al., 2019).

#### ATP-Binding Cassette Subfamily B Member 1

The ATP-binding cassette subfamily B member 1 (ABCB1), commonly known as P-glycoprotein or multidrug resistance protein 1 (Dean et al., 2001), is an efflux pump transporting compounds out of the cell (Chang, 2003). Initially known to be responsible for chemoresistance (Juliano and Ling, 1976; Sharom, 2008), the focus on its role in drug disposition has expanded from cytotoxic medications to include many other drugs (Leschziner et al., 2007).

There are more than 3,000 *ABCB1* variants, with SNPs 1236T > C (rs1128503), 2677T > G/A (rs2032582), and 3435T > C (rs1045642) being the three most studied (Wang et al., 2005). These SNPs have not been sufficiently characterized to establish a confident genotype-to-phenotype prediction, largely due to discordant study results. For example, studies of the rs1045642 SNP have reported an increase (Hitzl et al., 2001) as well as decrease (Nakamura et al., 2002) in ABCB1 expression.

The three SNPs mentioned above are in strong linkage disequilibrium, which leads to common haplotypes of 1236**C**:2677**G**:3435**C** and 1236**T**:2677**T**:3435**T** (Wang et al., 2005). These haplotypes are defined by two common star allele designations: *ABCB1^∗^1* and *ABCB1^∗^2*. However, the lack of standardization in referencing and assigning *ABCB1* haplotypes has resulted in inconsistent nomenclature (Hodges et al., 2011; Kalman et al., 2016), and therefore, assessment of the impact of *ABCB1* genetic variation on statin PK has been variable (Table 6).

**TABLE 6:** Summary ABCB1 studies.

Study design	Sample size	Ethnicity	*SNPs*	Analysis	Statins	Findings	Reference
Retrospective cohort	299	Caucasian	3435T > C (rs1045642)	SNPs	Atorvastatin Rosuvastatin	No significant association with the plasma concentration of atorvastatin and rosuvastatin.	(DeGorter et al., 2013)
Prospective cohort	758	Chinese Han	3435T > C (rs1045642)	SNPs	Rosuvastatin *N*-desmethyl rosuvastatin	No significant effects on the plasma concentration of rosuvastatin, rosuvastatin lactone and *N*-desmethyl rosuvastatin.	(Bai et al., 2019)
Case–control	Case: 33 Control: 33	Not reported	1236T > C (rs1128503) 3435T > C (rs1045642)	SNPs	Atorvastatin Rosuvastatin Simvastatin	Significantly higher risk of CK elevation in homozygotes 1236**TT** than heterozygotes (*p* < 0.05, OR = 4.67). Marginal significant effects on the risk of CK elevation for 3435**TT** compared to heterozygotes or 3435**CC** (*p* = 0.051).	(Ferrari et al., 2014)
Single-dose pharmacokinetic	28	Korean	2677T > G/A (rs2032582) 3435T > C (rs1045642)	Diplotype	Atorvastatin 2-hydroxy-atorvastatin	40% higher AUC of atorvastatin lactone in TT/TT group than GC/GC and GC/TT group (*p* = 0.028). No significant effects on the AUC of atorvastatin acid, 2-hydroxyatorvastatin acid and 2-hydroxyatorvastatin lactone.	(Lee et al., 2010)
Single-dose pharmacokinetic	12	Chinese	1236T > C (rs1128503) 2677T > G/A (rs2032582) 3435T > C (rs1045642)	SNPs	Pitavastatin	40% and 60% higher AUC (*p* = 0.004) and Cmax (*p* = 0.007) of pitavastatin acid in 2,677 **non-G carriers** than 2677**GT/GA/GG**. 20% higher Cmax of pitavastatin lactone in 2,677 **non-G carriers** than 2677**GT/GA/GG** (*p* = 0.038). No significant association between 1236T > C and 3435T > C with the PK of pitavastatin acid and lactone.	(Zhou et al., 2013a)
Single-dose randomized open label	133	Korean	1236T > C (rs1128503) 2677T > G/A (rs2032582) 3435T > C (rs1045642)	SNPs	Simvastatin	No significant effects on the AUC of simvastatin acid and simvastatin lactone.	(Choi et al., 2015)
Randomized crossover pharmacokinetic	12	Chinese	1236T > C (rs1128503) 2677T > G/A (rs2032582) 3435T > C (rs1045642)	SNPs Haplotype	Rosuvastatin	30% higher AUC of rosuvastatin in homozygotes 1236**TT** when compared with heterozygotes 1236**CT** (*p* = 0.007). 30% higher AUC of rosuvastatin in 2677 **non-G carriers** when compared with 2677**GT/GA/GG** (*p* = 0.012). 30% higher AUC of rosuvastatin in homozygotes 3435**TT** when compared with 3435**TC** and 3435**CC** (*p* = 0.012). 30% higher AUC of rosuvastatin in TTT/TTT when compared with non-TTT/TTT (*p* = 0.012).	(Zhou et al., 2013b)
Crossover pharmacokinetic	24	Finnish	1236T > C (rs1128503) 2677T > G/A (rs2032582) 3435T > C (rs1045642)	Haplotype	Simvastatin Atorvastatin 2-hydroxy-atorvastatin 4-hydroxy-atorvastatin	60% and 55% higher mean AUC of simvastatin acid (*p* = 0.039) and atorvastatin acid (*p* = 0.025) in TTT/TTT when compared with CGC/CGC. 50% and 42% higher mean AUC for 2-hydroxyatorvastatin acid (*p* = 0.018) and 4-hydroxyatorvastatin lactone (*p* = 0.034) in TTT/TTT when compared with CGC/CGC. No significant effects on the PK of simvastatin lactone, atorvastatin lactone and 2-hydroxyatorvastatin lactone.	(Keskitalo et al., 2008)
Crossover pharmacokinetic	20	Caucasian	1236T > C (rs1128503) 2677T > G/A (rs2032582) 3435T > C (rs1045642)	Haplotype	Fluvastatin Pravastatin Lovastatin Rosuvastatin	No association was found between haplotype CGC/CGC and TTT/TTT with the AUC of fluvastatin, pravastatin, lovastatin, and rosuvastatin.	(Keskitalo et al., 2009a)
Single-dose pharmacokinetic	26	Korean	1236T > C (rs1128503) 2677T > G/A (rs2032582) 3435T > C (rs1045642)	Haplotype	Simvastatin	No association was found between haplotype CGC/CGC and TTT/TTT with the AUC of simvastatin acid and lactone.	(Jiang et al., 2017)

**TABLE 7:** List of GWAS studies reviewed.

No	Study Title	Reference
1	SLCO1B1 variants and statin-induced myopathy—a genomewide study	(Link et al., 2008)
2	Genomewide Association Study of Statin-Induced Myopathy in Patients Recruited Using the United Kingdom Clinical Practice Research Datalink	(Carr et al., 2019)
3	A genome-wide association study of circulating levels of atorvastatin and its major metabolites	(Turner et al., 2020)
4	Association of common variants in the human eyes shut ortholog (EYS) with statin-induced myopathy: evidence for additional functions of EYS	(Isackson et al., 2011)

Similarly, the association between *ABCB1* genotypes and SIM is unclear. For the SNP 1236T > C (rs1128503), one study showed a significant (*p* = 0.049) association between the C allele and development of myalgia (Fiegenbaum et al., 2005). In contrast, a study conducted by Becker et al. (2010) showed no association of the 1236T > C SNP (rs1128503) and dose reduction or statin change during simvastatin or atorvastatin therapy. Similarly, no association was found in a study by Hermann et al. (2006), which compared 15 controls and 14 cases of atorvastatin-induced myopathy. These studies also analyzed SNPs 2677T > G/A (rs2032582) and 3435T > C (rs1045642). While no significant results were observed for these SNPs in two studies (Hermann et al., 2006; Becker et al., 2010), Fiegenbaum and colleagues (2005) (Fiegenbaum et al., 2005) reported that the SNPs 3435T > C (rs1045642) and 2677T > G/A (rs2032582) were significantly (*p* = 0.03) associated with myalgia development (Fiegenbaum et al., 2005). In addition, Morimoto et al. (2005) reported a significant association between the 2677T > G/A SNP (rs2032582) and simvastatin or atorvastatin-induced myopathy (*p* < 0.05). With respect to SNPs 3435T > C (rs1045642), the results reported by Fiegenbaum et al. (2005) were not replicated by two further studies. Hoenig et al. (2011) investigated the impact of SNPs 3435T > C (rs1045642) on the efficacy and safety of atorvastatin. Of 154 participants enrolled in the study, 10 reported myalgia. Analysis showed that the T allele of 3435T > C (rs1045642) was significantly (*p* = 0.043) associated with atorvastatin-induced muscle symptoms (Hoenig et al., 2011). Similarly, this observation was supported by Becker et al. (2010), who found that the T allele of 3435T > C (rs1045642) resulted in a higher risk of dose reduction or statin change during simvastatin or atorvastatin therapy. However, the association did not reach significance.

In addition to individual SNP analysis, Fiegenbaum et al. (2005) investigated the association of SIM with eight haplotypes. Only one haplotype (1236**T**:2677**non-G**:3435**T**) showed a significant association (*p* = 0.027).

The genetic variations of *ABCB1* have been reported to affect statin disposition. However, functional characterization of *ABCB1* SNPs is necessary to understand its impact on statin PK and subsequently the association with the risk of SIM.

#### ATP-Binding Cassette Subfamily G Member 2

ATP-binding cassette subfamily G member 2 (ABCG2) belongs to one of the seven subfamilies across the 48 ABC transporters in humans (Dean et al., 2001), with physiological roles in regulating the movement of substances across human tissues (Robey et al., 2009; Cuperus et al., 2014). To date, more than 200 substrates of ABCG2 have been identified including antibiotics, statins, and anticoagulants (Horsey et al., 2016; Heyes et al., 2018). The most extensively studied variant is a missense variant 421C > A (rs2231142). This SNP reduces the transporter activity of ABCG2 by enhancing proteasomal degradation, which leads to impaired protein expression (Yanase et al., 2006; Furukawa et al., 2009). Another common SNP is 34G > A (rs2231137). However, this SNP does not affect protein expression levels when compared with wild type (Imai et al., 2002; Kondo et al., 2004; Yanase et al., 2006; Tamura et al., 2007). Similarly, PharmGKB, an established pharmacogenomic online database, have reported no functional effects for this SNP (Fohner et al., 2017).

The statins, atorvastatin, fluvastatin, pitavastatin, pravastatin, and rosuvastatin are known substrates of ABCG2 (Fohner et al., 2017). Rosuvastatin is the most studied statin with respect to SNP 421C > A (rs2231142). In a PK study in 14 healthy Chinese participants, a single oral dose of 20 mg rosuvastatin showed a doubling in AUC of rosuvastatin in carriers of the A allele (*p* = 0.018) (Zhang et al., 2006). This was replicated in a bigger Chinese cohort consisting of 62 samples by Wan et al. (2015). Interestingly, the positive PK association between *ABCG2* 421C > A (rs2231142) with rosuvastatin in Chinese is well replicated in the European population. For example, a gene–dose effect was observed in a PK study on 32 healthy Finnish subjects. The AUC of rosuvastatin in homozygote A allele carriers was 50% (*p* = 0.004) and 60% (*p* < 0.001) higher than heterozygotes and non-carriers, respectively (Keskitalo et al., 2009b). This is supported by further studies, which reported a higher plasma concentration of rosuvastatin in carriers of the A allele (DeGorter et al., 2013; Birmingham et al., 2015). A recent study by Bai et al. (2019) investigated the impact of *ABCG2* 421C > A (rs2231142) on rosuvastatin and its metabolites: rosuvastatin lactone and *N*-desmethyl rosuvastatin. The AUC measured in A allele homozygotes was significantly higher when compared with heterozygotes and non-carriers for all three compounds (Bai et al., 2019). The association observed with rosuvastatin and *N*-desmethyl rosuvastatin was also supported by Lee et al. (2013).

The association between *ABCG2* 421C > A (rs2231142) and the AUC of statin is evident in other studies with respect to rosuvastatin (Zhou et al., 2013b; Liu et al., 2016; Kim et al., 2019), atorvastatin (Keskitalo et al., 2009b; Birmingham et al., 2015), and simvastatin acid (Birmingham et al., 2015; Choi et al., 2015). However, contrasting and negative findings have also been published. No association of rs2231142 with AUC was found for rosuvastatin (Kim et al., 2017), atorvastatin (DeGorter et al., 2013), pitavastatin (Ieiri et al., 2007; Oh et al., 2013; Zhou et al., 2013b), simvastatin lactone (Birmingham et al., 2015; Choi et al., 2015), lovastatin acid (Zhao et al., 2017), and lovastatin lactone (Zhao et al., 2017). Furthermore, no association between this SNP and CK elevation was reported in a study cohort taking either atorvastatin, rosuvastatin, or simvastatin (Ferrari et al., 2014). With respect to SIM, the findings were contrary. While there is a significant association between *ABCG2* 421C > A (rs2231142) and the PK of rosuvastatin, the results were not significantly associated with SIM. As an example, in a study of 758 participants, 51 patients developed myopathy after rosuvastatin treatment. No significant association was found between the 421C > A SNP (rs2231142) and SIM (Bai et al., 2019).

In contrast, Mirosevic and colleagues (2015) (Mirosevic Skvrce et al., 2015) genotyped and compared 60 patients who experienced dose-related ADRs with atorvastatin with 90 matching controls. The ADRs included myotoxicity and hepatotoxicity, where myotoxicity constituted a majority of the events. Carriers of the A allele were determined to have a 2.9-fold greater risk of developing dose-related atorvastatin ADRs (*p* = 0.016) (Mirosevic Skvrce et al., 2015). This effect was more prominent with fluvastatin, where the risk of ADRs associated with A allele carriers was 4.76-fold when compared with non–A-allele carriers (CC homozygotes) (*p* = 0.014) (Mirosevic Skvrce et al., 2013). However, this is a cohort targeted study where all of the samples studied were renal transplant patients taking fluvastatin. Therefore, interpreting and generalizing the results should be done with caution due to confounding factors such as the concurrent administration of immunosuppressants, which might be substrates or inhibitors of ABCG2 transporters.

As for 34G > A *ABCG2* SNP (rs2231137), Wan et al. (2015) reported a significantly higher AUC of rosuvastatin in homozygotes of the A allele (*p* < 0.01) among 62 healthy Chinese volunteers. However, this association with rosuvastatin was not replicated by Zhou et al. (2013b). Furthermore, no significant effects on pitavastatin acid and its lactone form were reported (Zhou et al., 2013a).

#### ATP-Binding Cassette Subfamily C Member 2

Adenosine triphosphate-binding cassette subfamily C member 2 (ABCC2) or multidrug resistance protein 2 is also a member of the wide ABC transporter family. Highly expressed in the liver, kidney, and intestine (Nguyen et al., 2013), this efflux membrane transporter holds important roles in drug disposition. Although all statins (simvastatin, pravastatin, pitavastatin, fluvastatin, atorvastatin, rosuvastatin, lovastatin) are substrates of ABCC2 (Ellis et al., 2013), PK studies on statins and genetic variation in the ABCC2 transporter are lacking. The most commonly studied *ABCC2* SNP with respect to statin disposition is 1249G > A (rs2273697). However, the functional characterization of this SNP is unclear, although it may impair the transporter function of glutathione and glucuronide-conjugated substrates (Kim et al., 2010; Megaraj et al., 2011).

Studies investigating the effect of the 1249G > A SNP (rs2273697) on the PK profile of statins reported no association between the variant allele and plasma concentration or AUC of rosuvastatin (DeGorter et al., 2013), atorvastatin (DeGorter et al., 2013), pravastatin (Niemi et al., 2004; Ho et al., 2007), and pitavastatin (Oh et al., 2013). A similar trend was observed with SIM. Becker et al. (2013) studied the impact of *ABCC2* genotypes on any dose reduction or statin switching events during simvastatin and atorvastatin therapy. With a sample of 1,014 subjects (789 simvastatin users, 225 atorvastatin users) from the Rotterdam study, they reported that the number of dose reduction or statin switching events significantly (*p* = 0.045) increased with the number of A alleles. However, this finding was not replicated in a separate statin-specific analysis on simvastatin and atorvastatin (Becker et al., 2013).

Another promising variant is *ABCC2* -24C > T (rs717620). Oh et al. (2013) reported a significant association between the AUC of pitavastatin with SNPs *ABCC2* -24C > T (rs717620). Participants homozygous for the T allele had a lower AUC of pitavastatin when compared with non–T-allele carriers (*p* = 0.028).

## Pharmacodynamics and Other Important Genetic Factors

To this point, genetic variation affecting the PK of statins has been discussed. However, there are other contributors reported to have a role in SIM. This includes proteins which are involved in the PD of statins, synthesis of muscle toxicity biomarkers, or immunologically associated ADRs. These are briefly discussed below.

### Glycine Amidinotransferase

*Glycine amidinotransferase* (*GATM*) gene encodes for the L-arginine:glycine amidinotransferase enzyme. This enzyme converts L-arginine into guanidinoacetate, a rate-limiting step in creatine synthesis (Wyss and Kaddurah-Daouk, 2000). The potential role of *GATM* genetic variations contributing to SIM was highlighted by Mangravite et al. (2013), this study explored expression quantitative trait loci (eQTL) in SIM cases. In this study, lymphoblastoid cell lines were exposed to simvastatin and the expression of various genes assessed. The change in expression of the *GATM* gene was strongly associated with statin exposure, and a further analysis of SNPs across the *GATM* locus revealed rs9806699 as being the most significantly associated differential eQTL (deQTL) after *in vitro* exposure to simvastatin. To test the relationship of this deQTL with SIM, an association analysis of rs9806699 was carried out in two patient cohorts, the first comparing 72 myopathy cases with 220 matched controls, and the second including 100 myopathy cases. A significant association was observed in both studies independently, and a meta-analysis of these two studies showed that the A allele of rs9806699 was significantly associated with a reduced risk of SIM (OR = 0.6, *p* = 6 × 10^–4^) (Mangravite et al., 2013).

However, these results have not been replicated. Luzum et al. (2015) genotyped 609 cases and 106 controls in a multicenter, case–control study. The analysis was conducted on three different arms: mild SIM, severe SIM, and combined mild and severe SIM groups. No significant associations were found between *GATM* and SIM in all three groups. This study also did a meta-analysis on related studies and reported a non-significant association between *GATM* and SIM (OR = 0.82, *p* = 0.072) (Luzum et al., 2015). This was supported by further research on 150 SIM cases and 587 statin-exposed control patients (Carr et al., 2014). No significant differences were found between the frequency of the A allele with myopathy (OR = 0.94, *p* = 0.68) or severe myopathy (OR = 0.94, *p* = 0.83). Further analysis in patients receiving only simvastatin (99 cases and 344 controls) did not reveal a significant association between either myopathy (OR = 1.12, *p* = 0.49) or severe myopathy (OR = 1.42, *p* = 0.24) with *GATM* (Carr et al., 2014). In addition, a case–control study of 175 cerivastatin-induced rhabdomyolysis cases and 645 statin users as controls found no association between *GATM* rs9806699 and the risk of rhabdomyolysis (OR = 1.01, *p* = 0.96) (Floyd et al., 2014).

There are several factors that might lead to the differing results observed for *GATM*. One, which has been discussed before, is the different definitions of myopathy and case inclusion criteria. Ethnicity is another important factor to consider; Bai et al. (2019) assessed SIM incidents in 758 Chinese rosuvastatin users with coronary artery disease. The A allele of *GATM* rs9806699 was reported to have a marginally protective effect on SIM (OR = 0.617, *p* = 0.024). Therefore, ethnicity-dependent effects should be considered when assessing the association of *GATM* with SIM (Bai et al., 2019).

### Coenzyme Q_2_

*Coenzyme Q_2_* (*COQ*_2_) is one of the many genes required for the biosynthesis of coenzyme Q_10_ (COQ_10_). At least 13 genes are involved in this pathway and *COQ*_2_ encodes for 4-hydroxybenzoate prenyl-transferase, which mediates the second last step in the reaction synthesis of COQ_10_ (Forsgren et al., 2004; Doimo et al., 2014). COQ_10_ or ubiquinone plays an important role in producing ATP, which drives the mitochondrial respiratory chain, pyrimidine biosynthesis, and apoptosis regulation (Doimo et al., 2014). As briefly mentioned in the pathogenesis of SIM, statins reduce COQ_10_ levels by up to 40% in both healthy volunteers and hypercholesteremic patients (Ghirlanda et al., 1993; Thompson et al., 2003). While several reviews and a clinical trial did not support the role of COQ_10_ supplementation in preventing SIM (Marcoff and Thompson, 2007; Schaars and Stalenhoef, 2008; Banach et al., 2015; Taylor et al., 2015), a recent meta-analysis on 12 randomized controlled trials concluded that COQ_10_ supplementation provided symptomatic relief for patients with SIM (Qu et al., 2018).

The two common SNPs investigated in the *COQ*_2_ gene are *COQ*_2_ A > G (rs6535454) and *COQ*_2_ G > A/C/T (rs4693075) (Oh et al., 2007). Oh et al. (2007) compared 133 European subjects on statin monotherapy who suffered from myotoxicity with 158 matched controls. They reported a significant association between statin intolerance and the SNPs rs6535454 (*p* = 0.047) and rs4693075 (*p* = 0.019). This was supported by Puccetti et al. (2010), who showed that the rs4693075 SNP was associated with atorvastatin or rosuvastatin-induced SIM. Subjects taking rosuvastatin and atorvastatin had a significantly (*p* < 0.001) higher risk (2.6- and 3.1-fold) of being statin intolerant with muscular symptoms and CK elevation, respectively (Puccetti et al., 2010). However, there are several studies that did not find a significant association between *COQ*_2_ SNPs and SIM (Carr et al., 2013; Hubacek et al., 2017; Ramakumari et al., 2018). The populations studied varied across these three reports from Caucasian (Carr et al., 2013), South Indian (Ramakumari et al., 2018) to Czech (Hubacek et al., 2017).

### Human Leukocyte Antigen and Leukocyte Immunoglobulin-Like Receptor Subfamily B5

Human leukocyte antigen (HLA) has been associated with human disease for decades with its role in body physiology, protective immunity, and disease-causing autoimmune reactivity (Dendrou et al., 2018). As mentioned previously, statin-induced anti–HMGCR-associated myopathy is a rare condition associated with statins. A significant association between HMGCR autoantibodies and *HLA* locus (*HLA-DRB1^∗^11*) has been reported by Limaye et al. (2015) with an OR of 50 (*p* < 0.0001). This is supported by Mammen et al. (2012), who studied 28 European and African American patients with HMGCR autoantibodies. When compared with 654 controls, *HLA-DRB1^∗^11* was identified in 70% and 18% of the European cases and controls, respectively (*p* = 1.2 × 10^–6^). Similarly, 88% and 21% out of the African cases and controls had *HLA-DRB1^∗^11* (*p* = 0.0002) (Mammen et al., 2012).

*Leukocyte immunoglobulin-like receptor subfamily B5* (*LILRB5*) gene is another immunogenic risk factor associated with SIM (Siddiqui et al., 2017). To date, only one variant (rs12975366 T>C), which is also referred to as 247Asp > Gly within the *LILRB5* locus, has been reported as being associated with statin intolerance and myalgia. A study in nearly 12,000 people from the Scottish GoDARTS cohort reported a twofold risk in developing general statin intolerance in participants homozygous for the T allele. A replication study conducted in the JUPITER trial showed an OR of 1.35 for developing myalgia (*p* = 0.04). A meta-analysis of the initial and replication studies showed a significant association of the homozygous T allele with a 1.5-fold higher risk of developing statin intolerance (*p* = 7 × 10^–5^) (Siddiqui et al., 2017).

## Other Genes

There are other genes not discussed here that have been reported to have an association with the risk of developing SIM. They include *apolipoprotein E* (*APOE*) (Hubáček et al., 2015b; Liu et al., 2017), *HMGCR* (Frudakis et al., 2007), *vitamin D receptor* (*VDR*) (Ovesjo et al., 2016), *serotonin receptors HTR3B* and *HTR7* (Ruano et al., 2007), *chloride channel protein 1* (*CLCN1*) (Neroldova et al., 2016), *eyes shut homolog* (*EYS*) (Isackson et al., 2011; Stranecky et al., 2016), *glycosyl transferase-like protein* (*LARGE*) (Stranecky et al., 2016), *ryanodine receptor 1* and *2* (*RYR1* and *RYR2*) (Marciante et al., 2011; Isackson et al., 2018), and *calcium voltage-gated channel subunit alpha 1S* (*CACNA1S*) (Isackson et al., 2018).

The genetic variations in these genes are not well studied, and their association with SIM is not widely replicated. Therefore, their roles as candidate genes in exploring SIM remain unclear.

## Discussion and Conclusion

For decades, statins have remained as an important drug in cardiovascular health. Despite emerging alternatives such as proprotein convertase subtilisin/kexin type 9 (PCSK9) inhibitors (Wang and Liu, 2019) and bempedoic acid (Laufs et al., 2019), statins as affordable and effective cholesterol-lowering agents are likely to remain as first-line in the treatment of hypercholesterolemia. In the era of personalized medicine, genomics offers promising approaches for research and development, especially in optimizing efficacy and reducing ADRs.

As reviewed, there are several genetic targets that may increase the risk of developing SIM. While all of the CYP enzymes discussed are involved in statin metabolism, it is likely that genetic variations in enzymes with major metabolism roles have a significant effect on statin disposition. They are CYP2C9, CYP3A4, and CYP3A5. As for phase II metabolism, the role of UGT in statin metabolism and SIM should be further explored. With respect to transporters, SLCO1B1, SLCO2B1, and ABCG2 are good candidates, whereas proper functional characterization of ABCB1 is necessary prior to applying any strong conclusions of their association with SIM.

While the discovery of rs4149056 in the *SLCO1B1* transporter gene is an example of a significant and initially striking finding, several further studies have failed to replicate this finding in different cohorts (Puccetti et al., 2010; Brunham et al., 2012; Carr et al., 2013; Danik et al., 2013; Hou et al., 2015; Hubáček et al., 2015a; Khine et al., 2016; Liu et al., 2017; Ramakumari et al., 2018). Similarly, the impact of genetic variation in pharmacogenes (*CYP-450*) and other genes (*GATM*, *CoQ*_2_, etc.) have been largely inconclusive. The ambiguity in findings shows that more research in elucidating the pharmacogenomic impact of these genes on SIM is warranted. Several factors may contribute to these discordant results, and the lack of clear and robust findings, which are listed below:

1) SIM has a complex and inexplicit pathogenesis.
2) SIM is heterogeneous and lacks standardized phenotype definition.
3) There are limited genetic association studies on statin-induced myalgia, which is the most commonly occurring form of SIM (e.g., Ruano et al., 2011; Siddiqui et al., 2017; Elam et al., 2017).
4) Metabolic enzymes exhibit substrate specificity (e.g., fluvastatin is a substrate of CYP2C9, but not CYP3A4).
5) Unclear functional characterization of SNPs in genes of interest (e.g., *ABCB1* gene).
6) The role of non-genetic factors in SIM (e.g., environmental influences and comorbidity).

Furthermore, an important covariate in genetic studies with SIM is ethnicity. Two examples discussed in this review are *SLCO1B1* rs4149056 with SIM (Liu et al., 2017) and the protective effect of *GATM* rs9806699 (Bai et al., 2019), both these studies investigated rosuvastatin-induced myotoxicity in a Chinese population. As discussed, Asian populations prescribed rosuvastatin have generally been reported having a higher plasma exposure of rosuvastatin compared to non-Finnish Europeans. This led to the FDA recommendation of reducing the starting dose of rosuvastatin by 50% in Asian patients (Lee et al., 2005; Liao, 2007). The higher plasma exposure in Asians is still unclear and not associated with genetic variation in *SLCO1B1* (Lee et al., 2005; Tomita et al., 2013; Birmingham et al., 2015). Because SIM is dose-related, plasma concentration exposure is one of the risk factors in developing SIM. Therefore, study results derived from a specific ethnic population should be replicated and validated before generalizing the findings to other populations.

Clearly, further research is required to understand the genetic association with SIM. One promising approach is the generation of a polygenic risk score (PRS). PRS is defined as an individualized risk burden, quantitatively measured from a range of common, intermediate and rare risk alleles, identified by large-scale genome sequencing in predisposing phenotype susceptibility (Chatterjee et al., 2016; Torkamani et al., 2018). In addition to that, PRS may be combined with clinical and lifestyle factors. Since its introduction in 2007 (Wray et al., 2007), PRS have been trialed by researchers for risk stratification to inform disease screening and therapeutic intervention. SIM may be a good candidate for PRS application because of its multifactorial causal mechanisms associated with genetic variation.

Ultimately, a clearly elucidated association will assist in the clinical implementation of statin pharmacogenetics. CPIC has published a clinical guideline on the management of patients who have specific *SLCO1B1* genetic variants, specifically with respect to statin prescription and dosing. A recent systematic review reported that genotype testing may influence the prescribing of statins among high-risk patients, but the impact of SIM or cardiovascular events was not reported (Vassy et al., 2019). Peyser et al. (2018) conducted a prospective randomized *SLCO1B1* genotype–guided statin therapy trial in 159 patients who stopped taking statins because of a history of statin myalgia. The study reported that when compared with the cohort receiving standard care, *SLCO1B1* pharmacogenetic testing improved statin reinitiation and lowering of LDL among identified *SLCO1B1^∗^5* carriers. However, there was no improvement on self-reported statin adherence (Peyser et al., 2018). Because SIM is one of the main factors leading to poor statin adherence and subsequently higher risk of cardiovascular events (De Vera et al., 2014), educating and providing patients with clear information along with *SLCO1B1* pharmacogenetic testing may improve statin adherence (Lansberg et al., 2018).

While it may be feasible to incorporate *SLCO1B1* pharmacogenetic testing clinically, further well-designed randomized controlled trials are necessary to investigate its clinical utility. A recent pragmatic trial conducted by Brunette et al. (2020) describes a promising approach. By referring to the PRagmatic-Explanatory Continuum Indicator Summary 2 (PRECIS-2) framework, statin-naive patients were randomized into two arms: with or without *SLCO1B1* rs4149056 genotype result available to healthcare providers through electronic health records. The clinical impact of *SLCO1B1* genotyping in reducing SIM was one of the outcomes measured. However, it was not reported in this publication (Brunette et al., 2020).

Although it is clear that the rs4149056 SNP is strongly associated with simvastatin-induced myopathy, the broader association of other genetic variation in pharmacogenes remains a challenge with respect to understanding their role in SIM. Despite a great deal of existing research, we may still be some way from the ability to apply genomic knowledge clinically to predict and ideally prevent the occurrence of SIM.

## Author Contributions

PK wrote the first draft of this article. SM and MK aided in the editing of this manuscript. PC provided clinical insight into this review and helped with the editing of the manuscript. All authors contributed to the article and approved the submitted version.

## Conflict of Interest

The authors declare that the research was conducted in the absence of any commercial or financial relationships that could be construed as a potential conflict of interest.

## Abbreviations

ABC: ATP-binding cassette
ABCB1: ATP-binding cassette subfamily B member 1
ABCC2: ATP-binding cassette subfamily C member 2
ADRs: adverse drug reactions
APOE: apolipoprotein E
ATP: adenosine triphosphate
AUC: area under the concentration–time curve
CACNA1S: calcium voltage-gated channel subunit alpha 1S
CK: creatine kinase
CLCN1: chloride channel protein 1
COQ_10_: coenzyme Q_10_
COQ_2_: coenzyme Q_2_
CPIC: Clinical Pharmacogenetics Implementation Consortium
CYP450: cytochrome P450
deQTL: differential expression quantitative trait loci
eQTL: expression quantitative trait loci
EYS: eyes shut homolog
FDA: Food and Drug Administration
GATM: glycine amidinotransferase
gnomAD: genome aggregation database
GoDARTS: Genetics of Diabetic Audit and Research
GWAS: genome-wide association studies
HLA: human leukocyte antigen
HLA-DRB1: human leukocyte antigen DR-isotope beta chain
HMG-CR: hydroxymethylglutaryl-coenzyme A reductase
HTR3B: 5-hydroxytryptamine receptor 3B
HTR7: 5-hydroxytryptamine receptor 7
JUPITER: Justification for the Use of Statins in Primary Prevention, An Intervention Trial Evaluating Rosuvastatin
LARGE: glycosyl transferase-like protein
LDL: low-density lipoprotein
LILRB5: leukocyte immunoglobulin-like receptor subfamily B5
OR: Odds ratio
PCSK9: proprotein convertase subtilisin/kexin type 9
PD: pharmacodynamics
PK: pharmacokinetics
PRIMO: Prediction of Muscular Risk in Observational Conditions
PRS: polygenic risk score
RYR1: ryanodine receptor 1
RYR2: ryanodine receptor 2
SIM: statin-induced myotoxicity
SLC: solute carrier
SLCO1B1: solute carrier organic anion transporter family member 1B1
SNPs: single nucleotide polymorphisms
SR: sarcoplasmic reticulum
SRM: statin-related myotoxicity
UGT: uridine 5 ′-diphospho (UDP)-glucuronosyltransferase
ULN: upper limit normal
VDR: vitamin D receptor.
